# Wide Swath and High Resolution Airborne HyperSpectral Imaging System and Flight Validation

**DOI:** 10.3390/s19071667

**Published:** 2019-04-08

**Authors:** Dong Zhang, Liyin Yuan, Shengwei Wang, Hongxuan Yu, Changxing Zhang, Daogang He, Guicheng Han, Jianyu Wang, Yueming Wang

**Affiliations:** Key Laboratory of Space Active Opto-Electronics Technology, Shanghai Institute of Technical Physics of CAS, Shanghai 200083, China; 15518289839@163.com (D.Z.); yuanliyin@mail.sitp.ac.cn (L.Y.); wswpp163@163.com (S.W.); yuhongxuan@mail.sitp.ac.cn (H.Y.); zhangchangxing@mail.sitp.ac.cn (C.Z.); hedaogang@mail.sitp.ac.cn (D.H.); hgc@mail.sitp.ac.cn (G.H.); jywang@mail.sitp.ac.cn (J.W.)

**Keywords:** wide swath, hyperspectral imager, high spatial resolution, high spectral resolution, airborne flight validation experiments

## Abstract

Wide Swath and High Resolution Airborne Pushbroom Hyperspectral Imager (WiSHiRaPHI) is the new-generation airborne hyperspectral imager instrument of China, aimed at acquiring accurate spectral curve of target on the ground with both high spatial resolution and high spectral resolution. The spectral sampling interval of WiSHiRaPHI is 2.4 nm and the spectral resolution is 3.5 nm (FWHM), integrating 256 channels coving from 400 nm to 1000 nm. The instrument has a 40-degree field of view (FOV), 0.125 mrad instantaneous field of view (IFOV) and can work in high spectral resolution mode, high spatial resolution mode and high sensitivity mode for different applications, which can adapt to the Velocity to Height Ratio (VHR) lower than 0.04. The integration has been finished, and several airborne flight validation experiments have been conducted. The results showed the system’s excellent performance and high efficiency.

## 1. Introduction

Spectral imagers can acquire not only 2D spatial image, but also spectral information of the target on Earth [1]. With the spectral information, many different targets can be identified and analyzed, which are usually quite difficult to realize using a traditional camera because of the similarity in RGB channel. Since JPL (Jet Propulsion Laboratory, Pasadena, CA, USA) developed the first spectral imagers AIS in 1983 [2], airborne hyperspectral imagers have played an important role in agricultural yield estimation, atmosphere component analyses, environmental monitoring, etc. In 1989, JPL developed a new airborne imaging spectrometer: The Airborne Visible/Infrared Imaging Spectrometer (AVIRIS) [3,4,5,6,7]. The new spectrometer covers a range from 0.4 μm to 2.4 μm using 224 channels. The spectral sampling interval is less than 10 nm. The swath width from U2 platform is 10.5 km, with instantaneous field of view (IFOV) of about 1 mrad. The AVIRIS system has been improved several times since it first became operational to provide more accurate data [4]. Meanwhile, many other typical airborne hyperspectral imaging systems have been developed, such as LEISA [8], AisaFENIX [9,10], OMIS [11], PHI, Hymap [12], CASI/SASI [13], etc. The main features are shown in Table 1.

Although these airborne hyperspectral imagers have been widely used in many fields [14,15,16,17], low spectral or spatial resolution or small field of view (FOV) restricting the working efficiency cannot meet the requirements of many modern applications. Areas such as environment pollution monitoring and precise agriculture require the imagers to have higher spectral and spatial resolution, larger dynamic range and wider FOV so that the pollution or crops can be investigated rapidly with high efficiency [18].

Under these circumstances, the new airborne hyperspectral imager project, Wide Swath and High Resolution Airborne Pushbroom Hyperspectral Imager (WiSHiRaPHI), was set up in 2013 [19,20]. Because the characteristic spectrum, which was often used to research surface materials, is mainly in the Visible and InfraRed band (Table 2), the wavelength range of the new hyperspectral imager is designed to cover 400–1000 nm. To make sure that the new imager has a high working efficiency, the FOV of WiSHiRaPHI is designed to be 40 degrees with a 0.125 mrad IFOV and 2.4 nm spectral sampling interval. 

## 2. WiSHiRaPHI System Introduction

**Figure 1:**
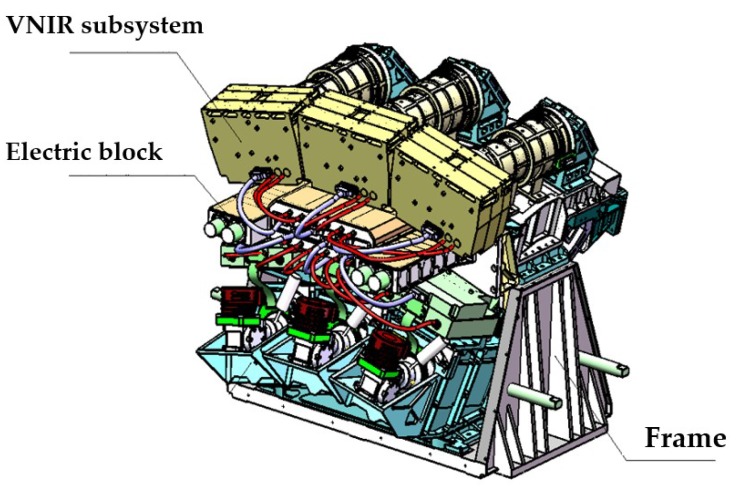
Sketch map of the Wide Swath and High Resolution Airborne Pushbroom Hyperspectral Imager (WiSHiRaPHI) system.

The WiSHiRaPHI system consists of three subsystems to acquire wide FOV. Every subsystem consists of fore optics, a spectrometer with planar blazed grating, electronics, three-axis platform and IMU (Inertial measurement unit), etc. The three subsystems work for the left, middle and right FOV, respectively. They are aligned on an arch frame to form the 40-degree FOV (Figure 1). The main features of the WiSHiRaPHI system are shown in Table 3.

### 2.1. Optical System

**Figure 2:**
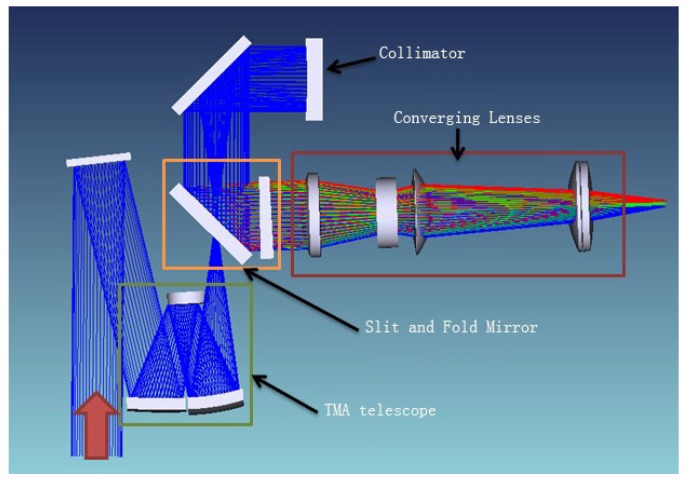
Optical system of WiSHiRaPHI.

The optical system consists of fore optics and a spectrometer subsystem. The fore optics adopts a three-mirror off-axis optics (TMA) design with compact structure, which facilitates system integration and image registration (Figure 2). The focal length of the fore optics is 128 mm, and F number is 3.8.

**Figure 3:**
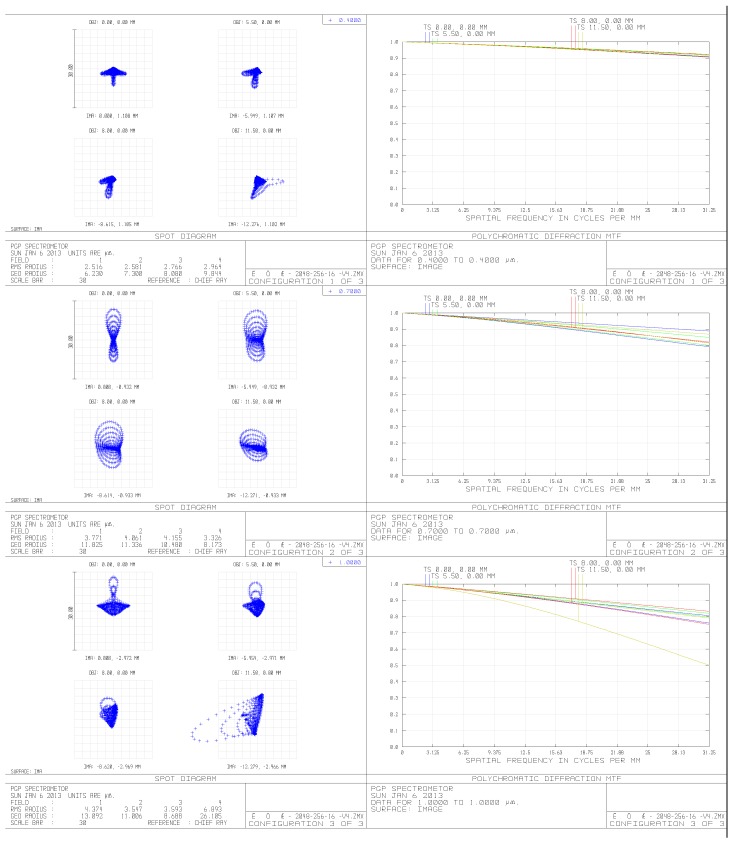
Modulation Transfer Function (MTF) curve and spot map of the optical system at 0.4 μm, 0.7 μm and 1 μm (left are spot maps, and right are MTF curve).

The spectrometer system consists of a collimator, a prism-grating dispersion element and an imaging lens. Through the primary optical system, the objects are imaged on the slit surface, dispersed by the prism-grating element and then converged on the photo-sensitive surface of the detector. Thus, the system has a low distortion and excellent Modulation Transfer Function (MTF) shown in Figure 3.

### 2.2. Photoelectric System

The photoelectric system is the core of the hyperspectral imager, which includes a CCD detector and camera electronics system. The CCD detector completes the function of photoelectric conversion, and the camera electronics system controls the running of the CCD detector.

**Figure 4:**
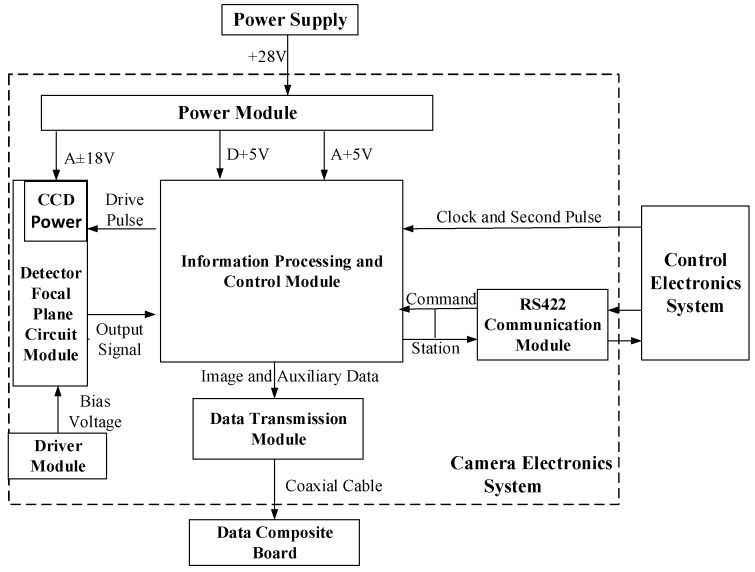
Electronic system block diagram.

The camera electronics system, shown in Figure 4, consists of a power module, a CCD interface module, a driver module, an information processing and control module, a data transmission module and an RS422 communication module. Thus, different imaging patterns with different spectral resolutions and spatial resolutions are provided. 

Taking detector scale, pixel size and frame frequency into account, a customized frame transfer CCD having a four-phase array back-illumination thinning frame transfer type detector with a total of 2048 × 256 pixels, a full well charge about 200 Ke^−^ and a pixel size of 16 μm × 16 μm was selected. The main performance parameters are shown in Table 4.

**Figure 5:**
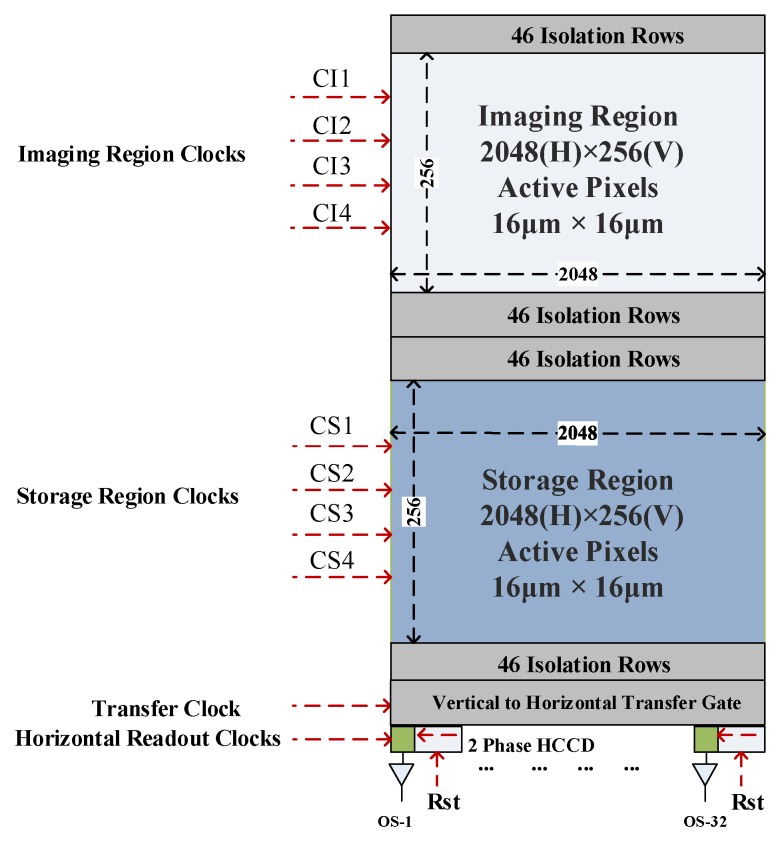
CCD structure diagram.

The CCD sensor architecture is shown in Figure 5. The CCD detector consists of three parts: The imaging region, the memory region and the horizontal read register. The resolution of the image region is 2048 (H) × 256 (V) pixels. On both sides of this region, along V direction, extra isolation rows are aligned, which may or may not be light-sensitive. CIx and CSx are the control signals for the imaging area and the memory area, and CRx is the pixel horizontal readout control signal. The sensor is operated by integrating photo-charges in the imaging region, after which it is transferred via rapid clocks into the storage region. While the storage region reads out the photo-charge into the horizontal CCD, the imaging region begins to integrate the next frame of photo-charge during this period. The sensor has a total of 34 taps, including 32 active taps and two dummy taps (one dummy tap on each side of the sensor. The dummy taps are not shown in the architecture). 

By controlling the timing of CS1, CS2, CS3, CS4 and RST, the photo-charges of adjacent pixels can be merged and realize online programming of spatial dimensions and spectral dimensions. If 256 spectral channels are adopted, the spectral resolution is 2.34 nm. When it corresponds to 64 channels, the spectral resolution is 9.32 nm. While satisfying the requirements of different applications, the pixel merger on spectral or spatial dimensions can also increase the effective size of the pixels, the sensitivity of the detector and the work frame frequency.

### 2.3. Block System

**Figure 6:**
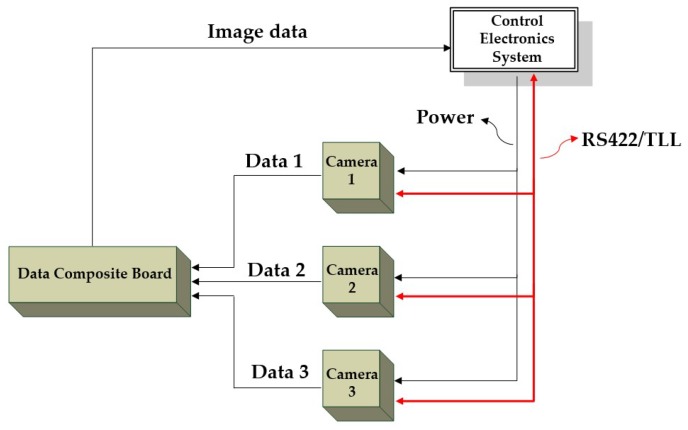
System block diagram.

The block system consists of three parts: Control electronics system, camera section (contains three cameras) and a data composite board, as shown in Figure 6. The control electronics system is designed to provide power supply, communication and data storage, which is the cerebrum of the block system. The camera section, as the core of the block system, includes detector driving, information acquisition and image data transmission. The main functions of the data composite board comprise data format conversion and data forwarding.

## 3. Laboratory Calibration

Accurate calibration of hyperspectral sensors is indispensable to the successful use of data from such sensors. Laboratory calibration is conducted after the major system is assembled in the laboratory to check whether or not the feature satisfies the assignment. The calibration mainly contains spectral calibration, radiometric calibration, MTF and Signal-Noise Ratio (SNR).

### 3.1. Spectral Calibration

**Figure 7:**
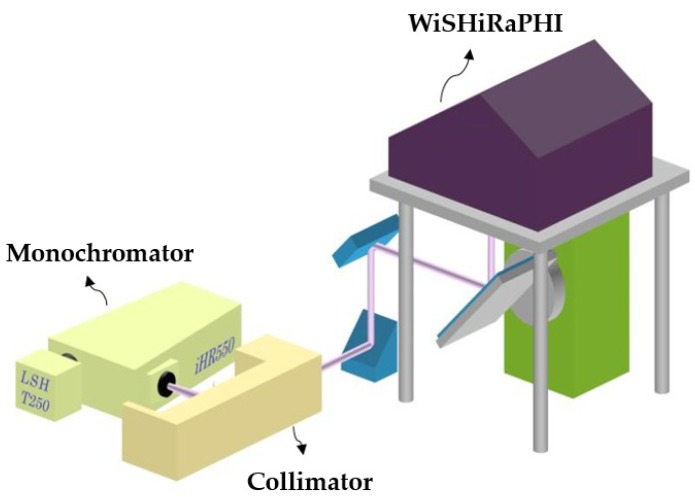
Full-field of view (FOV)-covered spectral calibration facility.

Spectral calibration is performed using monochromatic collimation light from a monochromator as the light source [21,22]. To calibrate the Spectral Response Function (SRF) of the whole FOV, a full-FOV-covered spectral calibration facility is designed to perform spectral calibration measurements, as shown in Figure 7.

**Figure 8:**
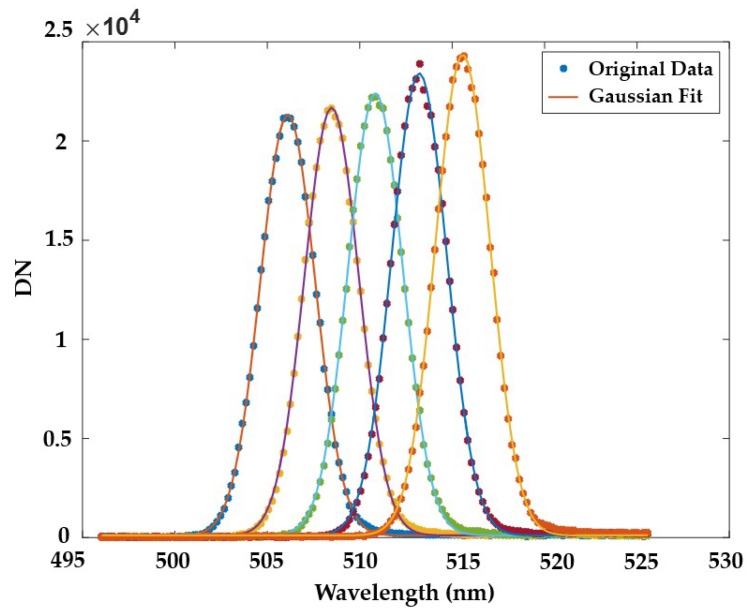
Spectral response curve of the left subsystem at Band 51–55 (mean full width at half maximum (FWHM) is 3.48 nm).

By changing the wavelength of the monochromatic collimation light each time, the spectral response curve of each channel is acquired (Figure 8). The SRF can be well-approximated by a Gaussian function with appropriate parameters as in Equation (1) [23].

(1) $$SRF\left(\lambda \right)=A_{0}+A_{1}\exp\left[-\frac{{\left(\lambda -\lambda_{0}\right)}^{2}}{2\sigma^{2}}\right]$$

where $\lambda_{0}$ is the center wavelength and full width at half maximum (FWHM) is calculated as $\mathrm{FWHM}=2\sqrt{2\ln2}\sigma$.

**Figure 9:**
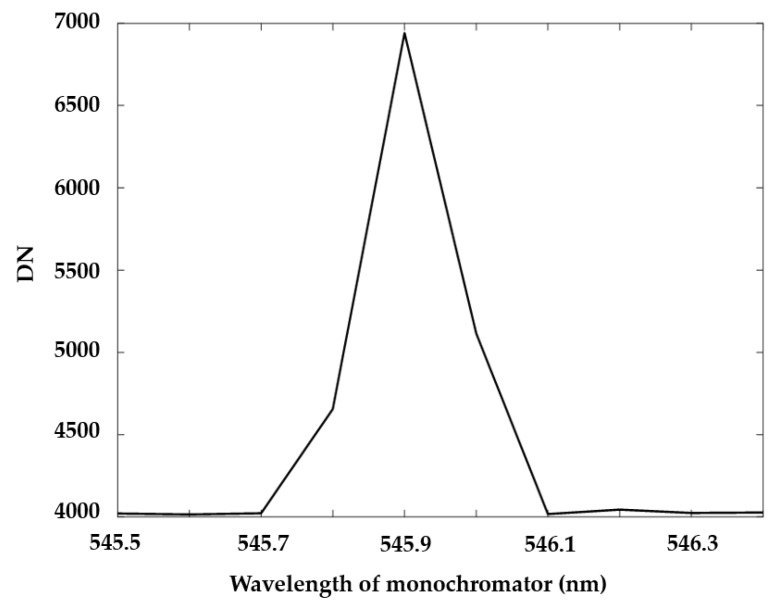
Monochromator’s response for 546 nm standard spectral line of mercury lamp (error of monochromator is 0.08 nm).

To make sure that the wavelength of out-light from the monochromator is accurate, a 546 nm standard spectral line of mercury lamp was used to correct the monochromator before scanning. By scanning the range around 546 nm at a step of 0.1 nm and calculating the barycenter of response curve, the accuracy of the monochromator would be below 0.1 nm (Figure 9).

### 3.2. Radiometric Calibration

Radiometric calibration contains relative radiometric calibration and absolute radiometric calibration. Relative radiometric calibration is conducted to correct the difference between different pixels in the same spectral channel. Absolute radiometric calibration is conducted to indicate the relationship between the pupil radiance and the response of the detector. Radiometric calibration is conducted using an integrating sphere and a standardized hyperspectral sensor.

Relative radiometric calibration accuracy is defined to indicate the error between the pixel’s response after relative radiometric calibration and the mean response of the same spectral channel. It is measured by setting the integrating sphere at different radiance levels and checking the response of the unstandardized hyperspectral sensor. Relative radiometric calibration accuracy is calculated as Equation (2).

(2) $$R_{R}\left(\lambda \right)=\underset{n=1\sim N}{mean}\left(\frac{\sqrt{\frac{1}{I-1}\sum_{i=1}^{I}{\left(DN_{i,n}\left(\lambda \right)-\bar{DN_{n}\left(\lambda \right)}\right)}^{2}}}{\bar{DN_{n}\left(\lambda \right)}}\right)$$

where $DN_{i,n}\left(\lambda \right)$ is the response of i-th pixel in λ channel at n-th radiance level after relative radiometric calibration, and $\bar{DN_{n}\left(\lambda \right)}$ is the mean response of all the pixels in λ channel at n-th radiance level.

**Figure 10:**
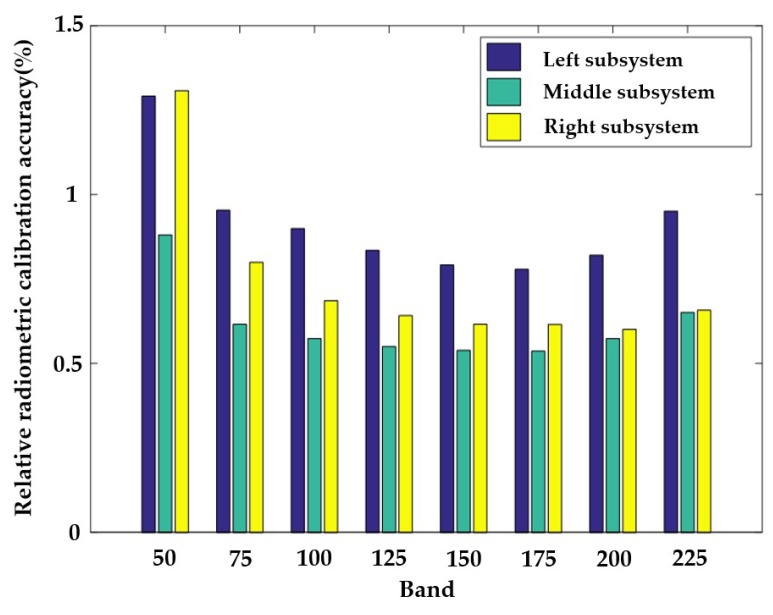
Relative radiometric calibration accuracy of three subsystems at Band 50, Band 75, Band 100, Band 125, Band 150, Band 175, Band 200, and Band 225.

The relative radiometric calibration accuracy of the hyperspectral imager depends on the worst $R_{R}\left(\lambda \right)$ for all λ. Thus, the relative radiometric calibration accuracy is 1.9%. Figure 10 shows the relative radiometric calibration accuracy of the three subsystems at eight different bands.

Absolute radiometric calibration accuracy is defined to indicate the error between the pixel’s response after relative radiometric calibration and the pupil radiance. It mainly contains three aspects: Unsteady error $R_{1}$, nonlinearity error $R_{2}$ and standardized hyperspectral sensor’s error $R_{3}$. The absolute radiometric calibration accuracy is measured using the response of the unstandardized hyperspectral sensor after relative radiometric calibration and it can be expressed as Equation (3):

(3) $$R_{A}=\sqrt{R_{1}^{2}+R_{2}^{2}+R_{3}^{2}}$$

Unsteady error indicates the stability of the sensor’s response for the same radiance. It is calculated as Equation (4):

(4) $$R_{1}=\underset{i,\lambda }{mean}\left(\underset{n=1\sim N}{\max}\left(\frac{\sqrt{\frac{\sum_{j=1}^{500}{\left(DN_{i,n}^{j}\left(\lambda \right)-\bar{DN_{i,n}\left(\lambda \right)}\right)}^{2}}{500}}}{\bar{DN_{i,n}\left(\lambda \right)}}\times 100\%\right)\right)$$

where $DN_{i,n}^{j}\left(\lambda \right)$ is the j-th response of i-th pixel in λ channel at n-th radiance level, and $\bar{DN_{i,n}^{}\left(\lambda \right)}=\frac{1}{500}\sum_{j=1}^{500}DN_{i,n}^{j}\left(\lambda \right)$.

Nonlinearity error is calculated as:

(5) $$R_{2}=\underset{i,\lambda }{mean}\left(\frac{RMSE_{i}\left(\lambda \right)}{\frac{1}{N}\sum_{n=1}^{N}DN_{i,n}\left(\lambda \right)}\times 100\%\right)$$

where $RMSE_{i}\left(\lambda \right)$ is the standard deviation of linear fitting residual error.

The standardized hyperspectral sensor’s error $R_{3}$ has a relationship with the standardized hyperspectral sensor, and usually is a constant. The absolute calibration accuracy of WiSHiRaPHI of the three subsystems is listed in Table 5.

### 3.3. MTF Determination

**Figure 11:**
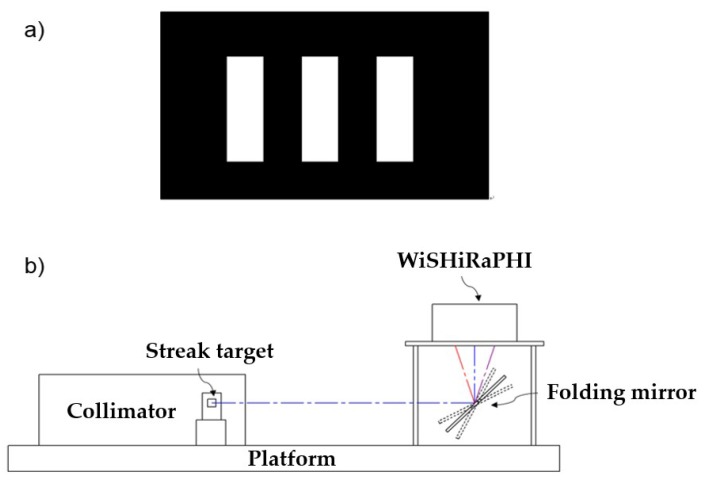
Sketch map of the streak target and MTF calibration system. (**a**) Shows the streak target and (**b**) shows the calibration system.

**Figure 12:**
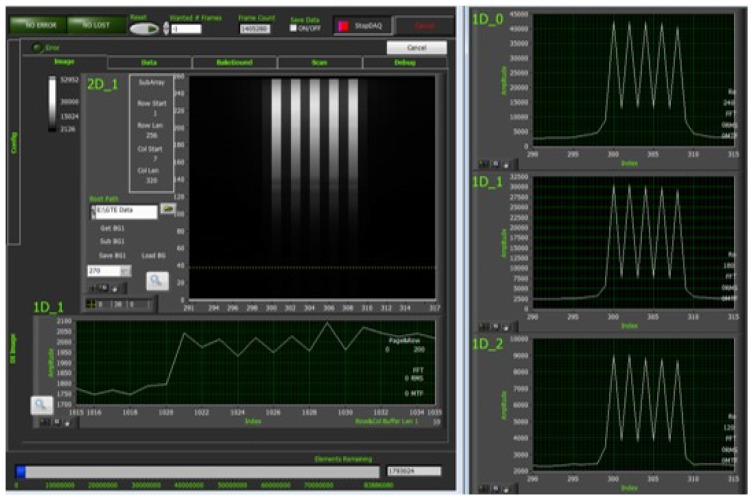
MTF calibration of three bands at the central FOV of high spectral mode (Band 120: MTF = 0.51, Band 180: MTF = 0.54, Band 240: MTF = 0.47).

**Figure 13:**
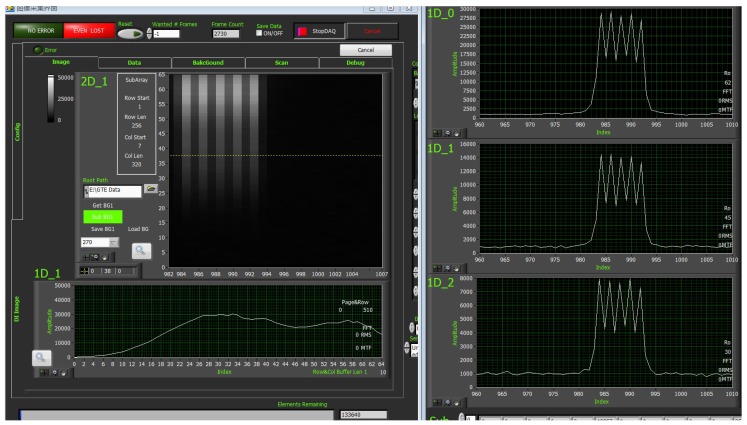
MTF calibration of three bands at the central FOV of high spatial mode (Band 30: MTF = 0.27, Band 45: MTF = 0.29, Band 62: MTF = 0.24).

MTF is one of the most important indicators to evaluate the image quality of a hypers-pectral imager. It indicates the modulation transfer characteristic of the imager to targets of different sizes, which will directly affect the sharpness of the hyperspectral sensing image in spatial direction. MTF calibration is conducted using a streak target at focal plane of collimator (Figure 11). The width of one streak is chosen to fit the focal length of collimator, thus the image on the detector just covers one pixel. With the response of the streak interdicting light, or not, MTF at Nyquist frequency is calibrated [24]. The MTF of high spectral mode and high spatial mode measured are shown in (Figure 12 and Figure 13).

(6) $$MTF_{Nyquist}=\frac{DN_{\max}-DN_{\min}}{DN_{\max}+DN_{\min}}\times \frac{\pi }{4}$$

where $DN_{\min}$ is the signal where the streak interdicts light (the dark signal between two bright signals) and $DN_{\max}$ is where the light passes through the streak. 

### 3.4. SNR Determination

**Figure 14:**
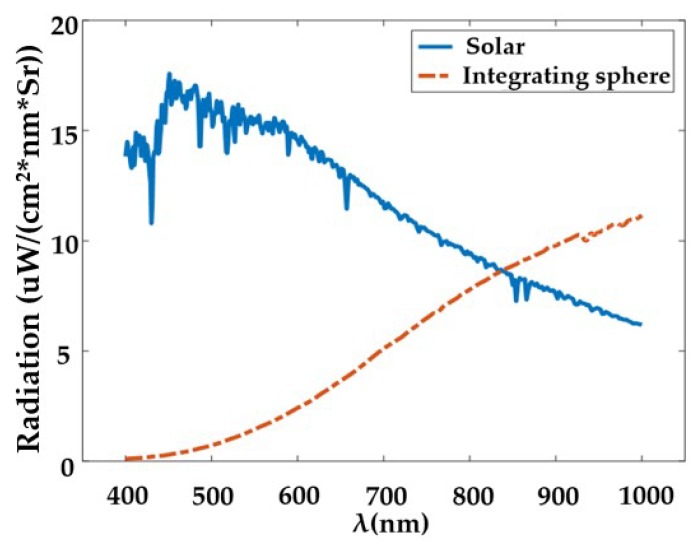
Spectral radiance curves of integrating sphere and solar.

SNR is the ratio of the equivalent charge of the output signal and the equivalent charge of the noise, and it is an important indicator to measure the detection sensitivity of the system. As with absolute radiometric calibration, SNR calibration is also conducted using the integrating sphere and standardized hypers-pectral sensor. Because the integrating sphere uses tungsten-halogen lamps as the light source, the spectral radiance curve of the integrating sphere is quite different from that of solar at all bands (Figure 14). Several radiance levels are used to make sure that the spectral radiance of the integrating sphere can approximate that of solar at several bands in proper radiance level. At each radiance level, 100 images are taken to calibrate SNR. The expression is shown as Equation (7).

(7) $$SNR_{i}\left(\lambda \right)=\frac{\frac{1}{100}\sum_{j=1}^{100}DN_{i}^{j}\left(\lambda \right)}{\sqrt{\frac{1}{99}\sum_{j=1}^{100}{\left(DN_{i}^{j}\left(\lambda \right)-\bar{DN_{i}^{dark}\left(\lambda \right)}\right)}^{2}}}$$

where $DN_{i}^{j}\left(\lambda \right)$ is the j-th response of i-th pixel in λ channel, and $\bar{DN_{i}^{dark}\left(\lambda \right)}$ is the mean background of i-th pixel in λ channel.

An extrapolation method is used to calculate the SNR in a laboratory to satisfy the condition in actual use. The specific formula for estimating the SNR from the actual measured SNR is Equation (8):

(8) $$SNR_{sun}\left(\lambda \right)=SNR_{measure}\left(\lambda \right)\cdot \sqrt{\frac{L_{sun}\left(\lambda \right)}{L_{measure}\left(\lambda \right)}}$$

where $L_{sun}\left(\lambda \right)$ is the radiometric of solar at λ wavelength, and $L_{measure}\left(\lambda \right)$ are the radiometric measures using integrating sphere.

**Figure 15:**
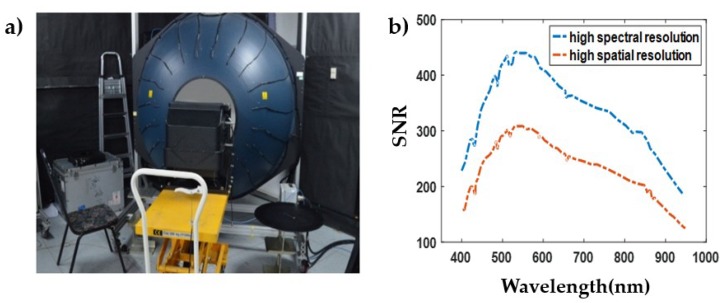
Scene of Signal-Noise Ratio (SNR) test (**a**) and the curve of SNR at central FOV (**b**).

Figure 15 shows the scene of the SNR test and the test result at central FOV of the system. The SNR curve indicates that WiSHiRaPHI has high sensitivity and large dynamic range.

## 4. Airborne Flight Validation Experiments

Several aerial flight experiments have been conducted to check the status of WiSHiRaPHI in Dongfang City of Hainan Province, Zhenjiang Port of Jiangsu Province, Xiong’an City of Hebei Province and Sansha City of Hainan Province, and lots of images with good quality were obtained [25].

**Figure 16:**
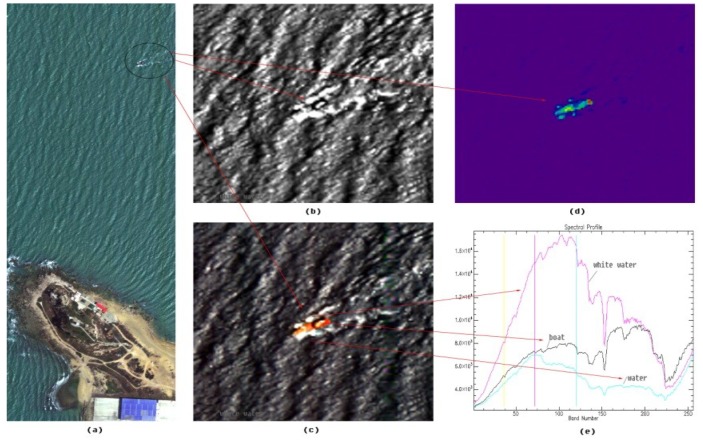
Hypers-pectral image of a port ((**a**) shows the RGB image of the coast, (**b**) and (**c**) show the images at two different spectral channels, (**d**) distinguishes the boat from the background using characteristic bands, and (**e**) shows the spectral curves of white water, boat and dark water).

Figure 16 shows the RGB synthesis image of a coast in Dongfang. A boat in the sea was easily distinguished from the oceanic background using some characteristic bands in hypers-pectral image because of the difference between the spectral curve of materials used in the boat and that of the oceanic background.

**Figure 17:**
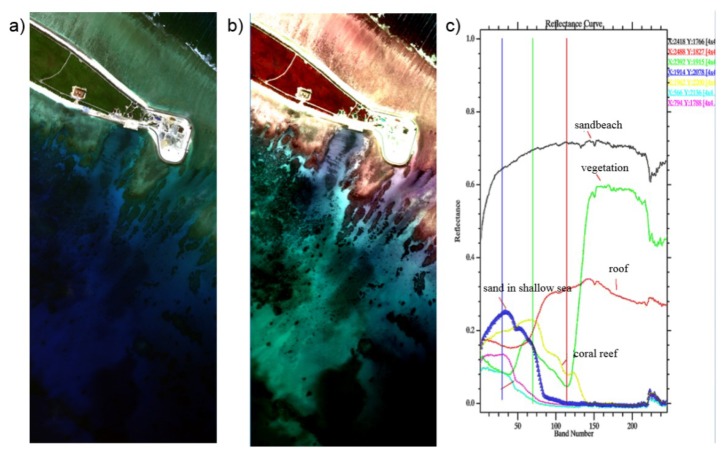
Hyperspectral image of island with atoll ((**a**) and (**b**) show the RGB image at different spectral channel, and (**c**) shows the reflectance curve of some typical targets).

Figure 17 shows the hypers-pectral image of an island with atoll. Figure 17a is an RGB image with R = 670 nm, G = 560 nm, B = 470 nm and Figure 17b with R = 600 nm, G = 480 nm, B = 430 nm. Figure 17c is the reflectance curve of some typical targets. By choosing the proper RGB channel, the target we needed was easily enhanced in the image.

**Figure 18:**
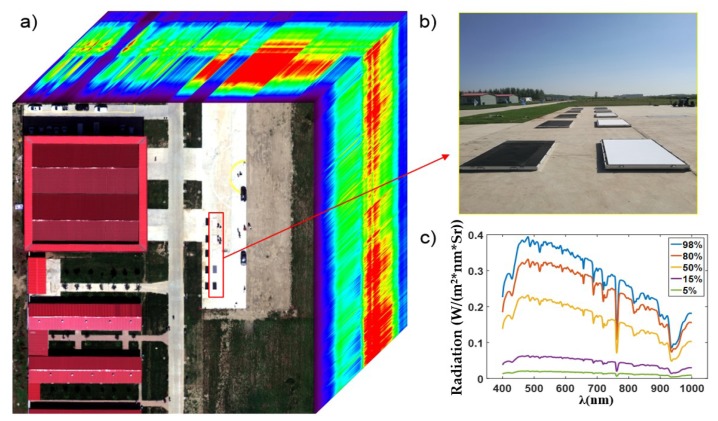
Hyperspectral image of diffuse reflectance targets ((**b**) is the picture of diffuse reflectance targets, and (**c**) shows the radiation curves of these diffuse reflectance targets).

A major application of the hyperspectral imager is to classify and measure the targets using the spectral radiance of the hyperspectral image. Because of the difference between the spectral radiance curve of the integrating sphere and that of solar, radiometric calibration in a laboratory was not precise enough. To increase the radiometric calibration accuracy, an air route for radiometric calibration was planned [26]. Five standard diffuse reflectance targets of different reflectance array were used along the trace to provide different radiance levels. An ASD hyperspectral sensor was used to detect the radiance value, when WiSHiRaPHI just flew over the diffuse reflectance targets (Figure 18). The image of each target should have at least covered 4×4 pixels to decrease the random error. Thus, the height and the speed of the air route have a relationship with the size of the diffuse reflectance targets and working frame frequency (Equation (9)).

(9) Ncross=Wcrossh⋅IFOVNalong=Walong⋅fvvh≤f⋅IFOV

where $N_{cross},N_{along}$ are the number of pixels that target covers across/along the trace, $W_{cross},W_{along}$ are the length of the target, v is the speed, h is the height and f is the working frame frequency.

**Figure 19:**
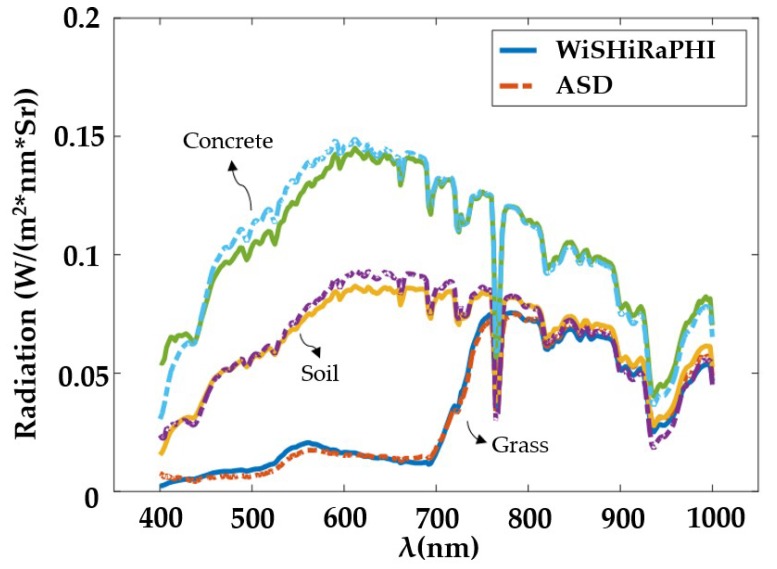
Hyperspectral image of diffuse reflectance targets.

To validate the radiometric calibration accuracy, several other targets around the diffuse reflectance targets were detected using ASD. The spectral radiance curves of these targets were both detected with ASD and WiSHiRaPHI, shown in Figure 19. The max residual error between ASD and WiSHiRaPHI was $0.02\;W/\left(m^{2}\cdot nm\cdot Sr\right)$.

**Figure 20:**
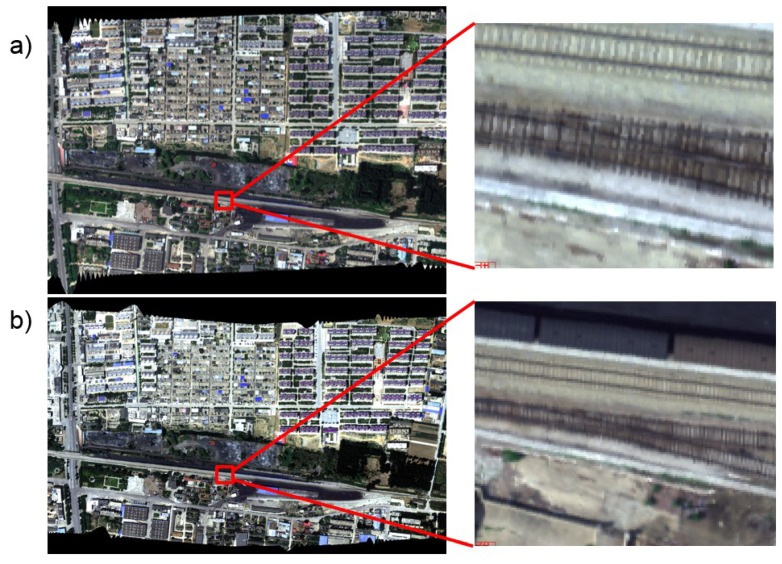
RGB synthesis image at high spatial/spectral resolution mode. (**a**) Is high spatial mode (instantaneous field of view (IFOV) = 0.125 mrad) and (**b**) is high spectral mode (IFOV = 0.25 mrad).

Figure 20 is the RGB synthesis image of Feicheng, Shandong at high spatial resolution mode and high spectral mode. The spatial resolution of high spatial resolution mode was 12.5 cm@1000 m and high spectral mode was 25 cm@1000 m. From the high spatial resolution image, many details such as venation of the rail and track of the car could be seen more clearly than in high spectral mode.

**Figure 21:**
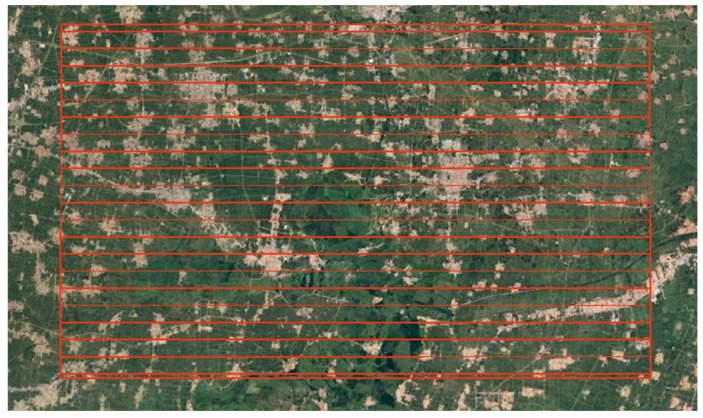
Air rout plan of Xiong’an City.

**Figure 22:**
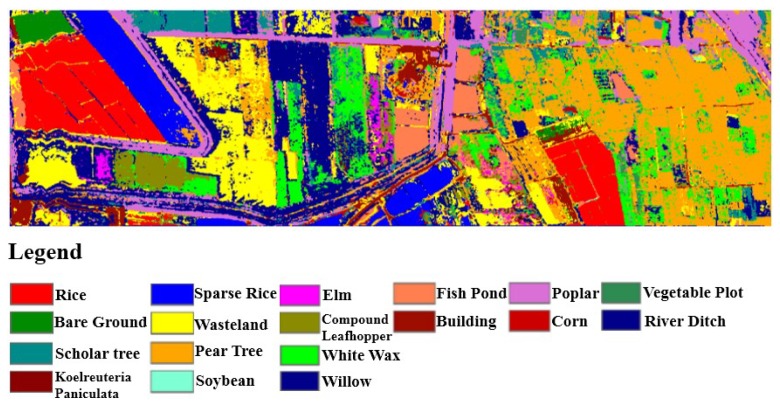
Land classification map.

In September 2018, WiSHiRaPHI had the opportunity of flying cover Xiong’an City. Thanks to the large FOV and dynamic range, the system covered an area about 28 km × 48 km just using twenty air routes at a height of 2100 m with the spatial resolution reaching 0.5 m in two days (Figure 21). Figure 22 shows the use of a plot of land in Xiong’an City using the image data obtained from the hyperspectral imager. With the spectral curve from the hyperspectral image, different elements were classified and identified, which shows the use of the hyperspectral imager in extracting crop growth information, monitoring crop growth and quality and protecting agricultural resources and environmental quality.

## 5. Conclusions

Hyperspectral imaging technology shows huge potential in many aspects because of its capacity to fuse traditional images with spectral images. The airborne hyperspectral imager has a high spatial resolution with high timeliness, which affords the application of airborne hyperspectral technology great convenience in environmental monitoring, agricultural resources investigation, mineral research, etc. The WiSHiRaPHI system is designed and integrated with the spectral range covering 400–1000 nm and spectral resolution better than 5 nm. The total FOV exceeding 40° and 0.25 mrad IFOV endow WiSHiRaPHI with high working efficiency and high spatial resolution. The weight below 20 kg with a power consumption of about 60 W means that the system could be installed on many platforms, such as ARJ-21, Y-12, etc. Several flight validation experiments were conducted, and a large amount of image data with good quality was obtained. This data has been used in actual business and meets the needs well. More flight missions have been arranged in the next year.

## Figures and Tables

**Table 1:** Main features of some typical airborne hyperspectral imaging systems.

	Spectral Range (nm)	Spectral Sampling Interval	Number of Channels	FOV (deg)	IFOV (mrad)
AVIRIS	400–2500	10 nm	224	30	1
LEISA	1000–2500	4–10 nm	432	19	2
AisaFENIX	380–2500	3–5 nm@380–970 nm 12 nm@970–2500 nm	448	32.3	1.4
OMIS	100–12,500	10 nm@0.46–1.1 μm 30 nm@1.1–1.7 μm 15 nm@2–2.5 μm 2 μm@3–5 μm 600 nm@8–12.5 μm	128	73	1.5/3
PHI	400–850	1.8 nm	244	21	1.5
Hymap	400–2500	10–20 nm	128	61.3	2 × 2.5
CASI/SASI	400–2500	2.4 nm/7.5 nm	96/200	40	0.49/0.698

**Table 2:** Characteristic spectral lines of typical material in Visible and InfraRed band.

Band (μm)	Characteristic Spectral Line	Band (μm)	Characteristic Spectral Line
0.46–0.48	Absorb of renieratene (high)	0.66–0.68	Valley of reflectance for most plant
0.50–0.52	Reflectance of chlorophyll (high)	0.70–0.72	Red edge of plant
0.54–0.56	Absorb of Fe^2+^, Fe^3+^	0.88–0.90	Peak of reflectance for plant, absorb of Fe^3+^
0.56–0.62	Absorb of phycoerythrin	0.92–0.94	Absorb of Fe^2+^

**Table 3:** Main features of the WiSHiRaPHI system.

Parameter	Index	Parameter	Index
Spectral Range (μm)	0.4–1.0	MTF	≥0.5
FOV	40°	VHR	0.02–0.04
Spectral Resolution (nm)	3.5/9.2, adjustable	Weight	≤20 Kg
Number of Channels	256/64, adjustable	Power Consumption	60 W
IFOV (mrad)	0.25/0.125, adjustable	Platform	ARJ-21, Y-12, and so on
SNR	≥500 (ρ = 0.3)		

**Table 4:** Parameters of the CCD detector.

Item	Parameters
Detector scale	2048 × 256, Frame transfer
Pixel size	16 μm × 16 μm
Number of output channels	32
Maximum pixel rate	25 MHz
Full well charge	≥200,000 e^−^
CCE	8 μV/e^−^
QE	≥61% @ 248 nm
Noise	≤55 e^−^

**Table 5:** Main features of the WiSHiRaPHI system.

Subsystems	R_1_	R_2_	R_3_	R_A_
Left	1.89%	0.29%	5.01%	5.36%
Middle	2.37%	0.28%	5.01%	5.55%
Right	1.45%	0.31%	5.01%	5.22%
